# Association of ghrelin with cardiometabolic risk factors in Iranian adolescents: the CASPIAN-III study

**DOI:** 10.15171/jcvtr.2016.23

**Published:** 2016-09-26

**Authors:** Ramin Heshmat, Gita Shafiee, Mostafa Qorbani, Fatemeh Azizi-Soleiman, Shirin Djalalinia, Mohammad Esmaeil Motlagh, Gelayol Ardalan, Zeinab Ahadi, Omid Safari, Saeid Safiri, Roya Kelishadi

**Affiliations:** ^1^Chronic Diseases Research Center, Endocrinology and Metabolism Population Sciences Institute, Tehran University of Medical Sciences, Tehran, Iran; ^2^Department of Community Medicine, Alborz University of Medical Sciences, Karaj, Iran; ^3^Endocrinology and Metabolism Research Center, Endocrinology and Metabolism Clinical Sciences Institute, Tehran University of Medical Sciences, Tehran, Iran; ^4^Department of Pediatrics, Child Growth and Development Research Center, Research Institute for Primordial Prevention of Non- Communicable Disease, Isfahan University of Medical Sciences, Isfahan, Iran; ^5^Development of Research & Technology Center, Deputy of Research and Technology, Ministry of Health and Medical Education, Tehran, Iran; ^6^Department of Pediatrics, Ahvaz Jundishapur University of Medical Sciences, Ahvaz, Iran; ^7^Department of Pediatrics, Alborz University of Medical Sciences, Karaj, Iran; ^8^Managerial Epidemiology Research Center, Department of Public Health, School of Nursing and Midwifery, Maragheh University of Medical Sciences, Maragheh, Iran

**Keywords:** Cardiovascular Risk Factors, Ghrelin, Anthropometric Measures, Metabolic Syndrome

## Abstract

***Introduction: ***Current evidence suggests that ghrelin could contribute to the development of metabolic syndrome (MetS) in adults, but limited experience exists in adolescents. This study aims to explore the association of ghrelin levels with the MetS components among Iranian adolescents.

***Methods: ***In this case-control study, 32 adolescents with MetS and 148 healthy controls were selected randomly from the childhood and Adolescence Surveillance and Prevention of Adult Non communicable disease (CASPIAN-III) study. MetS was defined according to the Adult Treatment Panel III (ATP III) criteria modified for children and adolescents. Anthropometric measures (including body mass index [BMI], waist circumference [WC] and waist to height ratio [WHtR]), blood pressure (BP) and biochemical data (including fasting blood sugar [FBS], triglyceride [TG], high-density lipoprotein cholesterol [HDL-C], low-density lipoprotein cholesterol [LDL-C], total cholesterol [TC] and gerlin) were measured.

***Results:*** Total ghrelin level was significantly higher in students without MetS compared to those with MetS (748.89 ± 85.04 vs. 728.72 ± 90.36 [pg/mL]; *P* < 0.001). Significant negative correlations were seen between ghrelin levels and BMI, WC, WHtR, TG, and TC. Ghrelin had also relatively strong inverse correlations with FBS (r = −0.59, *P*< 0.001), LDL-C (r = −0.56, *P* < 0.001), and positive correlation with HDL-C (r = 0.60, *P* < 0.001). Compared with the children with MetS, in those without MetS, ghrelin was significantly associated with HDL-C and LDL-C. A decreasing trend was observed in the mean ghrelin level across increasing number of MetS components (*P* for trend <0.001).

***Conclusion:*** We observed a relationship between ghrelin concentration and MetS components in adolescents.

## Introduction

Metabolic syndrome (MetS) is characterized by a cluster of abnormalities like insulin resistance, obesity, atherogenic dyslipidemia and hypertension. MetS is related to higher risk of chronic renal diseases, cardiovascular diseases, type II diabetes and mortality^1,2^ Although the prevalence of MetS depends on the criteria used in its definition, worldwide increasing rate of MetS has been of great interest recently.^3^ It is estimated that 20%-30% of adults suffer from MetS worldwide which is expected to increase in the future ^1^. NHANES 2001-2006 showed an 8.6% prevalence of MetS in adolescents.^4^ The prevalence of MetS was 10.1% in 2006 among Iranian adolescents.^5^ Increasing the incidence of MetS in adolescents appears to be associated with the growing epidemic of obesity, poor dietary habits, inactivity and sedentary lifestyle, and the social environment encouraging unhealthy behaviors.^6^

Ghrelin, also known as lenomorelin (INN), is predominantly produced by the gastrointestinal tract and mostly is involved in the regulation of energy balance.^7,8^ Ghrelin is regulated by nutritional status, blood glucose and insulin level, diet components, some hormones like leptin, somatostatin, thyroid hormone, testosterone, gut hormones, and parasympathetic nerves.^9^ This peptide is involved in controlling food intake, weight, insulin secretion, and gastric motility.^10,11^ It acts as an insulin secretagogue but the mechanism of its effects on fat metabolism is less clear.^12^ Current evidences suggest that ghrelin could contribute to the development of MetS in adults,^13,14^ but few studies has been studied this association among adolescents and to the best of our knowledge, no study to date has been conducted on Iranian adolescents. Considering recent increasing prevalence of MetS worldwide and addressing the knowledge gap in adolescents, we aimed to explore the association between ghrelin levels and the components of MetS among Iranian adolescents.

## Materials and Methods

The present study was conducted in the subsample of the third national surveillance program, entitled “Childhood and adolescents of surveillance and prevention of adult non-communicable disease” (CASPIAN- III) study, conducted between 2009- 2010. Participants of the CASPIAN III study were 5088 students aged 10–18 years that were selected using multistage random cluster sampling from urban and rural areas of 27 provinces of Iran. Detailed protocol and methods published elsewhere^15^ and herein we describe it in brief.

In this case-control study, 32 adolescents with MetS and 148 healthy controls which were selected randomly from the CASPIAN- III study. In the present study after including 32 eligible cases (students with MetS) 148 sex and age matched students without MetS were selected from the CASPIAN III study. Mets was defined according to the Adult Treatment Panel III (ATP III) criteria modified for children and adolescents.

Based on the standard data collection protocol, demographic, clinical, and biochemical measures were gathered from participants.^15,16^ Demographic characteristics included family history of chronic diseases (hypertension, dyslipidemia, diabetes, and obesity), parental education levels (total years of schooling), possession of a private car and house, dietary behaviors, and physical activity level.

Assessment of anthropometric measures and blood pressure (BP) were carried out following standard protocols by trained experts using calibrated instruments.^15,16^

Height (Ht) and weight (Wt) were measured, without shoes and with light clothing to the nearest 0.1 unit of measure (cm and kg). Body mass index (BMI) was calculated as weight (kg) divided by the square of height (m^2^).^13,14^ Waist circumference (WC) was measured midway between the lateral lower rib margin and the iliac crest at the end of normal expiration (cm). Abdominal obesity was defined as waist to height ratio (WHtR) more than 0.5.^17^

After a 12-hour overnight fasting, venous blood samples were drawn from all study participants and delivered the same day to the laboratory. Using auto-analyzers, fasting blood sugar (FBS), total cholesterol (TC), high-density lipoprotein cholesterol (HDL-C), and triglycerides (TG) were detected enzymatically. HDL-C was measured after dextran sulfate-magnesium chloride precipitation of non-HDL-C.

According to the Friedewald formula, low-density lipoprotein cholesterol (LDL-C) was calculated in serum samples with TG≤ 400 mg/dL. For the current sub-study, ghrelin was determined using the enzyme- linked immunosorbent assay (ELISA) kit (Abcam, USA). Intra and inter-assay coefficient of variation were 10 and 12%, respectively. Blood levels of ghrelin estimated as pmol/l.^18^

### Statistical analysis

Kolmogorov-Smirnov test was used to evaluate distribution of continuous variables for normality. Data are expressed either as frequencies (%) or as mean ± standard deviation (SD). Differences between groups were tested using *t* test and the Pearson chi-square test. Pearson correlation was applied to determine the correlation between ghrelin and cardiometabolic parameters. Analysis of covariance (ANCOVA) was used to evaluate the adjusted mean of ghrelin levels and cardio-metabolic risk factors. Adjustment was performed for sex, age, and body mass index (for elevated WC, only age and sex were adjusted). All statistical analysis was performed by SPSS (version 16.0, SPSS Inc., Chicago, Illinois) and *P*‏ values <0.05 were considered statistically significant.

## Results

In this case-control study, the mean ± SD of age was 14.31 ± 2.59 years with no significant difference between the groups with and without MetS. In term of gender, overall 53.1% and 48.6% of students in case and control group were male which was not statistically significant between groups (p: 0.27). Evaluation of anthropometric indicators showed that students with MetS had significant higher levels of weight (55.06 ± 17.81 vs. 42.41 ± 16.53 kg), BMI (21.85 ± 4.86 vs. 17.93 ± 4.28 kg/m^2^), WC (75.50 ± 13.71 vs. 64.51 ± 9.20 cm), and WHtR (0.48 ± 0.07 vs. 0.42 ± 0.05 cm) (*P* < 0.001 for all indicators).

Systolic and diastolic BP and FBS did not differ between the two groups. Except for HDL-C, which was significantly higher in participants without MetS (66.53 ± 20.79 vs. 44.06 ± 13.78 mg/dL), all other CVD risk factors including TG, LDL-C, and TC higher in MetS^+^ group (*P* < 0.001). Total ghrelin level was significantly higher in students without MetS compared to students with MetS (748.89 ± 85.04 vs. 728.72 ± 90.36 pg/ml; *P* < 0.001) (Table 1).

**Table 1:** Characteristics of adolescents with and without metabolic syndrome: the CASPIAN- III study

	**Total (n= 180)**	**MetS – (n= 148)**	**MetS + (n= 32)**	**P value**
Age (year)	14.31 ± 2.59	14.19 ± 2.64	14.91 ± 2.32	0.16
Weight (kg)	44.65 ± 17.40	42.41 ± 16.53	55.06 ± 17.81	<0.001
Height (cm)	152.11 ± 14.12	151.07 ± 14.46	156.94 ± 11.49	0.02
BMI (kg/m^2^)	18.63 ± 4.62	17.93 ± 4.28	21.85 ± 4.86	<0.001
WC (cm)	66.46 ± 10.94	64.51 ± 9.20	75.50 ± 13.71	<0.001
WHtR	0.43 ± 0.05	0.42 ± 0.05	0.48 ± 0.07	<0.001
SBP (mm Hg)	100.57 ± 14.22	99.68 ± 12.87	105.23 ± 19.50	0.07
DBP (mm Hg)	64.02 ± 10.11	63.91 ± 9.83	64.54 ± 11.62	0.77
FBS (mg/dL)	96.18 ± 27.63	93.47 ± 29.70	108.75 ± 5.51	0.004
TG (mg/dL)	100.53 ± 47.40	91.61 ± 37.26	143.10 ± 65.26	<0.001
HDL-C (mg/dL)	62.53 ± 21.49	66.53 ± 20.79	44.06 ± 13.78	<0.001
LDL-C (mg/dL)	75.89 ± 32.77	71.39 ± 32.36	96.90 ± 26.17	<0.001
TC (mg/dL)	152.23 ± 36.79	148.20 ± 37.53	170.75 ± 26.56	<0.001
Ghrelin (pg/ml)	728.72 ± 90.36	748.89 ± 85.04	635.50 ± 44.19	<0.001

Abbreviations: BMI, body mass index; WC, waist circumference; WHtR: waist- to -height ratio; SBP, systolic blood pressure; DBP, diastolic blood pressure; HDL-C, high-density lipoprotein cholesterol; TG, triglycerides; FBS, fasting blood sugar; LDL-C, low density lipoprotein cholesterol; TC: total cholesterol

The correlation between ghrelin values with cardio-metabolic risk factors are presented in Table 2. Significant negative associations were seen between ghrelin levels and BMI, WC, WHtR, TG, and TC. Ghrelin had also relatively strong correlations with FBS (r = −0.59, *P* < 0.001), LDL-C (r = −0.56, *P* < 0.001), and HDL-C (r = 0.60, *P* < 0.001).

**Table 2:** Correlation of ghrelin levels with cardiometabolic risk factors: the CASPIAN- III study

	**MetS – (n=148)**		**MetS + (n= 32)**		**Total (n= 180)**	
	**r**	**P value**	**r**	**P value**	**r**	**P value**
BMI (kg/m^2^)	-0.09	0.27	0.04	0.82	-0.22	0.003
WC (cm)	-0.02	0.81	0.05	0.78	-0.19	0.01
WHtR	-0.09	0.31	0.006	0.97	-0.23	0.002
SBP (mm Hg)	-0.08	0.38	-0.33	0.10	-0.15	0.05
DBP (mm Hg)	-0.03	0.77	-0.21	0.29	-0.05	0.52
HDL-C (mg/dL)	0.56	<0.001	-0.27	0.14	0.60	<0.001
TG (mg/ dL)	-0.04	0.63	-0.12	0.54	-0.24	0.001
FBS (mg/ dL)	-0.60	<0.001	0.43	0.02	-0.59	<0.001
LDL-C (mg/ dL)	-0.53	<0.001	-0.25	0.19	-0.56	<0.001
TC (mg/ dL)	-0.27	0.001	-0.45	0.01	-0.36	<0.001

Abbreviations: MetS: metabolic syndrome; BMI, body mass index; WC, waist circumference; WHtR: waist- to -height ratio; SBP, systolic blood pressure; DBP, diastolic blood pressure; HDL-C, high-density lipoprotein cholesterol; TG, triglycerides; FBS, fasting blood sugar; LDL-C, low density lipoprotein cholesterol; TC: Total Cholesterol

Compared with the children with MetS, in those without MetS, ghrelin was significantly associated with HDL-C and LDL-C. Moreover, we observed significant correlations between ghrelin and TC and FBS in both groups.

**Figure 1:**
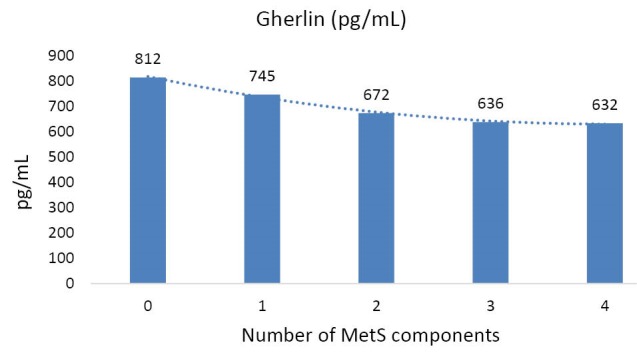
Mean of ghrelin concentration according to number of metabolic syndrome components: the CASPIAN- III study

As shown in Figure 1, a decreasing trend was observed in mean of ghrelin level with increasing the number of MetS components (*P* for trend <0.001).

Crude and adjusted means of ghrelin based on MetS and its single components are presented in Table 3. The crude and adjusted mean of ghrelin was significantly lower in students with MetS (*P* < 0.001). Those with increased levels of FBS, LDL, WC, TG and decreased HDL-C concentration had also lower ghrelin (*P* < 0.001 for all indicators).

**Table 3:** Association of ghrelin levels with cardiometabolic risk factors in crude and adjusted model: the CASPIAN III study

**Cardiometabolic risk factors**	**Crude model**			**Adjusted Model** ^ 1 ^		
	**Mean**	**SD**	***P *** ** value** ^ 2 ^	**Mean**	**SD**	***P *** ** value** ^ 3 ^
MetS -	748.89	85.04	<0.001	747.50	84.40	<0.001
MetS +	635.50	44.19		640.70	45.18	
Normal FBS	810.79	31.04	<0.001	810.66	32.97	<0.001
Elevated FBS	646.66	42.90		647.20	41.80	
Normal TC	733.30	90.12	0.03	731.87	89.23	0.09
Elevated TC	685.16	82.25		694.94	83.45	
Normal TG	741.57	85.93	0.02	739.75	85.28	0.04
Elevated TG	709.46	93.99		710.48	96.99	
Normal BP	730.97	90.14	0.49	730.14	92.38	0.61
Elevated BP	719.11	92.07		721.36	96.17	
Normal LDL	749.20	85.27	<0.001	747.31	85.56	<0.001
Elevated LDL	665.46	75.81		670.65	75.81	
Normal HDL-C	743.77	88.17	<0.001	741.39	86.43	<0.001
Low HDL-C	643.46	43.45		645.03	44.76	
Normal WC	735.71	90.20	0.002	733.16	92.10	0.001
Elevated WC	665.85	65.40		664.77	66.31	

Abbreviations: WC, waist circumference; BP, blood pressure; HDL-C, high-density lipoprotein cholesterol; TG, triglycerides; FBS, fasting blood Sugar; LDL-C, low density lipoprotein cholesterol; TC, Total Cholesterol. ^1^ Adjusted for sex, age and body mass index (for elevated WC only age and sex are adjusted); ^2^Are resulted from *t* test; ^3^Are resulted from ANCOVA test.

## Discussion

In our study, fasting ghrelin level was inversely associated with MetS components and serum HDL was positively associated with serum ghrelin among adolescents. Serum LDL and HDL were the only MetS components which significantly correlated with ghrelin level in adolescents with MetS, but not in the healthy controls. We also found a trend of decreasing ghrelin level with increasing the number of diagnostic criteria of MetS.

Some researchers have found ghrelin levels to be decreased in adults with MetS.^19,20^ As mentioned before, ghrelin is involved in glucose and lipid metabolism. Considering these functions, lower concentration of ghrelin in our patients with MetS proposes a role for this hormone in metabolic diseases.

An inverse association between ghrelin and anthropometric measurements is demonstrated in older adults and children.^21,22^ However, after including insulin or insulin sensitivity in multiple linear regression analyses to evaluate independent associations of anthropometric and metabolic parameters with ghrelin levels, BMI was no longer an independent predictor of ghrelin.^23^ Pacifico et al showed that WC, as an indicator of visceral fat, is more reliable than BMI and body fat mass in prediction of ghrelin level in children with MetS.^22^ Visceral fat had been shown an independent significant predictor of ghrelin level even after adjustment for insulin and BMI in premenopausal women.^24^ This may be related to the ability of visceral fat for secreting substances like free fatty acids, tumor necrosis factor-α, and leptin that are ghrelin inhibitors.^24^

Previous studies have reported an inverse association between ghrelin and BP, TG, FBS, and TC.^25,26^ Our findings confirm the relationship between single features of MetS and ghrelin. These relationships cannot be attributed to BMI, because lower level of ghrelin in adolescents with MetS in comparison with controls remained significant after it was adjusted. Ukkola et al also reported remaining the significant association between HDL and FBS with ghrelin after adjustment for BMI.^20^ Insulin can play role of a mediator. It seems that there is a negative association between ghrelin and insulin concentrations in adults.^23,27^ In children, total ghrelin have been shown to be positively associated with insulin resistance, independently of anthropometric and metabolic variables.^22^ It has shown that hyperinsulinemia may result in decreasing circulating ghrelin independently of glucose.^28^ Interestingly in our study, LDL and HDL had significant correlations with ghrelin in children without MetS. Unfortunately, in our study homeostatic model assessment of insulin resistance (HOMA-IR) was not assessed which can justify this finding.

We revealed when the number of components of MetS increased, a rising trend in ghrelin was appeared. This finding is supported by previous studies.^21,29^ It has shown that as the number of components of MetS increased, a progressive elevation in plasma acylated ghrelin and a decrease in plasma desacyl ghrelin were observed.^30^ Desacyl ghrelin is the major circulating form and acts as an antagonist of acylated ghrelin.^31^ Barazzoni et al finding suggested that the balance of the ratio of acylated to desacyl ghrelin affects insulin action in MetS.^32^

Some of the limitations of this study include small number of adolescents with MetS and not measuring insulin and acylated and des-acyl ghrelin. Further studies are necessary to distinguish the different effects of acylated and des-acyl ghrelin on MetS components.

In conclusion, we found a relationship between ghrelin concentration and MetS components in adolescents. The only difference between adolescents with and without MetS in terms of the association between ghrelin and MetS components was related to serum LDL and HDL that had significant correlations with ghrelin in controls. Ghrelin level decreased with an increase in number of MetS diagnostic criteria. Large cohorts are needed to clarify the contribution of ghrelin to the development of MetS.

## Acknowledgments

The authors are thankful of the large team working on this study and all participants in different provinces.

## Ethical approval

The study was conducted according to the declaration of Helsinki (Seoul, 2008). Ethical approval was given by the ethical committees and other relevant national and provincial regulatory organizations. Written informed consent and verbal acquiescence were obtained from parents and adolescents, respectively.

## Competing interests

All authors declare no competing financial interests exist.

## References

[1] Grundy SM (2008). Metabolic syndrome pandemic. Arterioscler Thromb Vasc Biol.

[2] Lee CC, Lee RP, Subeq YM, Wang CH, Fang TC, Hsu BG (2008). Fasting serum total ghrelin level inversely correlates with metabolic syndrome in hemodialysis patients. Arch Med Res.

[3] Ford ES, Giles WH (2003). A comparison of the prevalence of the metabolic syndrome using two proposed definitions. Diabetes Care.

[4] Johnson WD, Kroon JJ, Greenway FL, Bouchard C, Ryan D, Katzmarzyk PT (2009). Prevalence of risk factors for metabolic syndrome in adolescents: National Health and Nutrition Examination Survey (NHANES), 2001-2006. Arch Pediatr Adolesc Med.

[5] Esmaillzadeh A, Mirmiran P, Azadbakht L, Etemadi A, Azizi F (2006). High prevalence of the metabolic syndrome in Iranian adolescents. Obesity (Silver Spring).

[6] Zimmet P, Alberti KG, Kaufman F, Tajima N, Silink M, Arslanian S (2007). The metabolic syndrome in children and adolescents–an IDF consensus report. Pediatr Diabetes.

[7] Sakata I, Sakai T. Ghrelin Cells in the Gastrointestinal Tract. Int J Pept 2010; 2010: 945056. 10.1155/2010/945056. 10.1155/2010/945056 PMC2925405 20798855

[8] Burger KS, Berner LA (2014). A functional neuroimaging review of obesity, appetitive hormones and ingestive behavior. Physiol Behav.

[9] Korbonits M, Goldstone AP, Gueorguiev M, Grossman AB (2004). Ghrelin—a hormone with multiple functions. Front Neuroendocrinol.

[10] Van Der Lely AJ, Tschop M, Heiman ML, Ghigo E (2004). Biological, physiological, pathophysiological, and pharmacological aspects of ghrelin. Endocr Rev.

[11] Cummings DE, Shannon MH (2003). Roles for ghrelin in the regulation of appetite and body weight. Arch Surg.

[12] Smith RG, Thorner MO. Ghrelin in Health and Disease.Springer Science & Business Media; 2012.

[13] Kelishadi R, Marashinia F, Heshmat R, Motlagh M-E, Qorbani M, Taslimi M (2013). First report on body image and weight control in a nationally representative sample of a pediatric population in the Middle East and North Africa: the CASPIAN-III study. Arch Med Sci.

[14] Khashayar P, Heshmat R, Qorbani M, Motlagh ME, Aminaee T, Ardalan G (2013). Metabolic Syndrome and Cardiovascular Risk Factors in a National Sample of Adolescent Population in the Middle East and North Africa: The CASPIAN III Study. Int J Endocrinol.

[15] Kelishadi R, Heshmat R, Motlagh ME, Majdzadeh R, Keramatian K, Qorbani M (2012). Methodology and early findings of the third survey of CASPIAN study: a national school-based surveillance of students’ high risk behaviors. Int J Prev Med.

[16] Group WMGRS (2006). WHO Child Growth Standards based on length/height, weight and age. Acta Paediatr Suppl.

[17] Kelishadi R, Motlagh ME, Bahreynian M, Gharavi MJ, Kabir K, Ardalan G (2015). Methodology and early findings of the assessment of determinants of weight disorders among Iranian children and adolescents: The childhood and adolescence surveillance and prevention of adult Noncommunicable Disease-IV study. Int J Prev Med.

[18] Cummings D, Purnell J, Frayo R, Schmidova K, Wisse B, Weigle D (2001). A preprandial rise in plasma ghrelin levels suggests a role in meal initiation in humans. Diabetes.

[19] Chu MC, Cosper P, Orio F, Carmina E, Lobo RA (2006). Insulin resistance in postmenopausal women with metabolic syndrome and the measurements of adiponectin, leptin, resistin, and ghrelin. Am J Obstet Gynecol.

[20] Ukkola O, Pöykkö SM, Antero Kesäniemi Y (2006). Low plasma ghrelin concentration is an indicator of the metabolic syndrome. Ann Med.

[21] Langenberg C, Bergstrom J, Laughlin GA, Barrett-Connor E (2005). Ghrelin and the metabolic syndrome in older adults. J Clin Endocrinol Metab.

[22] Pacifico L, Poggiogalle E, Costantino F, Anania C, Ferraro F, Chiarelli F (2009). Acylated and nonacylated ghrelin levels and their associations with insulin resistance in obese and normal weight children with metabolic syndrome. Eur J Endocrinol.

[23] Purnell JQ, Weigle DS, Breen P, Cummings DE (2003). Ghrelin levels correlate with insulin levels, insulin resistance, and high-density lipoprotein cholesterol, but not with gender, menopausal status, or cortisol levels in humans. J Clin Endocrinol Metab.

[24] Sondergaard E, Gormsen L, Nellemann B, Vestergaard E, Christiansen JS, Nielsen S (2009). Visceral fat mass is a strong predictor of circulating ghrelin levels in premenopausal women. Eur J Endocrinol.

[25] Pöykkö SM, Kellokoski E, Hörkkö S, Kauma H, Kesäniemi YA, Ukkola O (2003). Low plasma ghrelin is associated with insulin resistance, hypertension, and the prevalence of type 2 diabetes. Diabetes.

[26] Kelishadi R, Hashemipour M, Mohammadifard N, Alikhassy H, Adeli K (2008). Short‐and long‐term relationships of serum ghrelin with changes in body composition and the metabolic syndrome in prepubescent obese children following two different weight loss programmes. Clin Endocrinol.

[27] McLaughlin T, Abbasi F, Lamendola C, Frayo RS, Cummings DE (2004). Plasma ghrelin concentrations are decreased in insulin-resistant obese adults relative to equally obese insulin-sensitive controls. J Clin Endocrinol Metab.

[28] Flanagan DE, Evans ML, Monsod TP, Rife F, Heptulla RA, Tamborlane WV (2003). The influence of insulin on circulating ghrelin. Am J Physiol Endocrinol Metab.

[29] Pöykkö S, Ukkola O, Kauma H, Kellokoski E, Hörkkö S, Kesäniemi Y (2005). The negative association between plasma ghrelin and IGF-I is modified by obesity, insulin resistance and type 2 diabetes. Diabetologia.

[30] Rodríguez A, Gomez-Ambrosi J, Catalan V, Becerril S, Sainz N, Gil MJ (2010). Association of plasma acylated ghrelin with blood pressure and left ventricular mass in patients with metabolic syndrome. J Hypertens.

[31] Gauna C, Delhanty PJ, Hofland LJ, Janssen JA, Broglio F, Ross RJ (2005). Ghrelin stimulates, whereas des-octanoyl ghrelin inhibits, glucose output by primary hepatocytes. J Clin Endocrinol Metab.

[32] Barazzoni R, Zanetti M, Ferreira C, Vinci P, Pirulli A, Mucci M (2007). Relationships between desacylated and acylated ghrelin and insulin sensitivity in the metabolic syndrome. J Clin Endocrinol Metab.

